# Impact of short-term, pharmacological CARM1 inhibition on skeletal muscle mass, function, and atrophy in mice

**DOI:** 10.1152/ajpendo.00047.2023

**Published:** 2023-07-26

**Authors:** Erin K. Webb, Sean Y. Ng, Andrew I. Mikhail, Derek W. Stouth, Tiffany L. vanLieshout, Anika L. Syroid, Vladimir Ljubicic

**Affiliations:** Department of Kinesiology, Faculty of Science, McMaster University, Hamilton, Ontario, Canada

**Keywords:** arginine methylation, autophagy, exercise, mitochondria, PRMT

## Abstract

Coactivator-associated arginine methyltransferase 1 (CARM1) catalyzes the methylation of arginine residues on target proteins critical for health and disease. The purpose of this study was to characterize the effects of short-term, pharmacological CARM1 inhibition on skeletal muscle size, function, and atrophy. Adult mice (*n* = 10 or 11/sex) were treated with either a CARM1 inhibitor (150 mg/kg EZM2302; EZM) or vehicle (Veh) via oral gavage for 11–13 days and muscle mass, function, and exercise capacity were assessed. In addition, we investigated the effect of CARM1 suppression on unilateral hindlimb denervation (DEN)-induced muscle atrophy (*n* = 8/sex). We report that CARM1 inhibition caused significant reductions in the asymmetric dimethylation of known CARM1 substrates but no change in CARM1 protein or mRNA content in skeletal muscle. Reduced CARM1 activity did not affect body or muscle mass, however, we observed a decrease in exercise capacity and muscular endurance in male mice. CARM1 methyltransferase activity increased in the muscle of Veh-treated mice following 7 days of DEN, and this response was blunted in EZM-dosed mice. Skeletal muscle mass and myofiber cross-sectional area were significantly reduced in DEN compared with contralateral, non-DEN limbs to a similar degree in both treatment groups. Furthermore, skeletal muscle atrophy and autophagy gene expression programs were elevated in response to DEN independent of CARM1 suppression. Collectively, these results suggest that short-term, pharmacological CARM1 inhibition in adult animals affects muscle performance in a sex-specific manner but does not impact the maintenance and remodeling of skeletal muscle mass during conditions of neurogenic muscle atrophy.

**NEW & NOTEWORTHY** Short-term pharmacological inhibition of coactivator-associated arginine methyltransferase 1 (CARM1) was effective at significantly reducing CARM1 methyltransferase function in skeletal muscle. CARM1 inhibition did not impact muscle mass, but exercise capacity was impaired, particularly in male mice, whereas morphological and molecular signatures of denervation-induced muscle atrophy were largely maintained in animals administered the inhibitor. Altogether, the role of CARM1 in neuromuscular biology remains complex and requires further investigation of its therapeutic potential in muscle-wasting conditions.

## Graphical abstract

**Figure:**
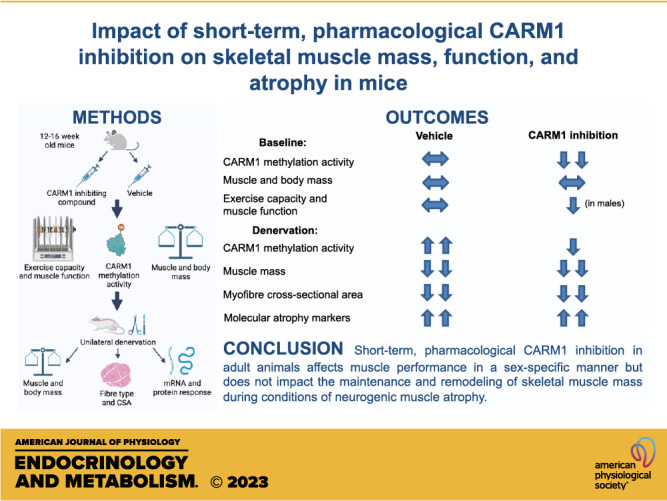

## INTRODUCTION

Coactivator-associated arginine methyltransferase 1 (CARM1) belongs to a family of enzymes known as protein arginine methyltransferases (PRMTs) that catalyze the transfer of methyl groups onto arginine residues of target proteins. Specifically, CARM1 (also known as PRMT4) catalyzes the addition of a single methyl group onto a terminal nitrogen atom of arginine to synthesize a monomethylarginine (MMA) modification and/or a second methyl group to create an asymmetric dimethylarginine (ADMA) mark. The addition of methyl groups to arginine residues on histone and nonhistone proteins may modify their physical and chemical properties to ultimately alter their activity and/or subcellular localization (1, 2). Arginine methylation occurs in human cells at the same frequency as other better understood, posttranslational modifications such as phosphorylation and ubiquitination (3) and is required for several cellular processes (1, 2). In fact, the whole body genetic deletion of CARM1 results in perinatal lethality in mice (4, 5). Moreover, in a cohort of over 141,000 participants, zero loss-of-function mutations in CARM1 were identified, which suggests that CARM1 is required for survival in humans as well (6, 7). Collectively, these data demonstrate that CARM1 plays critical roles in cell biology.

We recently demonstrated that in skeletal muscle, the prevalence of protein arginine methylation occurs on par with the extent of serine and threonine phosphorylation and lysine ubiquitination (8), posttranslational modifications that are much more studied and better understood to affect muscle phenotype maintenance and plasticity (9–11). Furthermore, we observed that CARM1 skeletal muscle-specific knockout (mKO) mice exhibited altered transcriptomic and arginine methylproteomic signatures within their skeletal muscles, as well as molecular and functional outcomes that suggested remodeled skeletal muscle contractile and neuromuscular junction characteristics, which presaged decreased exercise tolerance (8). Thus, CARM1 has emerged as an important regulator of skeletal muscle plasticity (12). This is not surprising since *1*) CARM1 is the most highly expressed PRMT transcript in rodent (13) and human (14) skeletal muscle, and *2*) CARM1 interacts with master regulators of skeletal muscle phenotype AMP-activated protein kinase (AMPK) and peroxisome proliferator-activated receptor-γ coactivator-1α (PGC-1α; 15, 16) and alters the activity of the AMPK-PGC-1α signaling axis (8, 15).

Given its role in skeletal muscle remodeling, regeneration, and repair (12), understanding CARM1 expression and function are of interest in the context of skeletal muscle wasting and weakness. We (15, 17) and others (18) have previously found that denervation (DEN) augments muscle CARM1 expression and methyltransferase activity concomitant with expected neurogenic disuse-induced muscle loss. Interestingly, in adult mice where skeletal muscle CARM1 content is transiently knocked down, as well as in CARM1 mKO animals, there is a preservation of muscle mass post-DEN and mitigation of the molecular atrophy program (15, 18). These findings were timely, as several small molecule PRMT inhibitor compounds are currently undergoing preclinical and clinical trials for various types of cancers (19, 20). However, the impact of pharmacological CARM1 inhibition on skeletal muscle biology is unknown. Thus, the purpose of the present study was to characterize the effects of CARM1 methyltransferase inhibition in adult mice (21, 22) on skeletal muscle mass, function, and atrophy. We hypothesized that global, pharmacological CARM1 inhibition would modestly impair skeletal muscle function, as well as attenuate the loss of mass and activation of atrophic signaling during neurogenic-disuse skeletal muscle atrophy.

## METHODS

### Animals

Young, (12–16-wk old) healthy female and male mice of C57BL6J/129 background were housed in an environmentally controlled room and provided food (standard chow diet) and water ad libitum. Following experiments, mice were euthanized via cervical dislocation, and the gastrocnemius (GAST), soleus (SOL), quadriceps (QUAD), tibialis anterior (TA), triceps (TRI), and extensor digitorum longus (EDL) muscles, as well as liver, were harvested. GAST, QUAD, TA, and TRI muscles were weighed and immediately flash frozen in liquid nitrogen, whereas SOL and EDL muscles were weighed and mounted in optimum cutting temperature compound (OCT; Fisher Scientific; Hampton, NH) and frozen in isopentane cooled in liquid nitrogen. All tissues were stored at −80°C until analysis. All mice were housed and cared for according to the Canadian Council on Animal Care guidelines in the McMaster Central Animal Facility.

### Pharmacological Inhibition of CARM1

Male and female littermates were randomized into either vehicle (Veh) or EZM2302 (EZM; Epizyme, Cambridge, MA) treatment groups. The Veh [0.5% sodium carboxymethyl cellulose (Sigma Aldrich; St. Louis, MO) in ddH_2_O] and EZM (150 mg/kg) solutions were prepared weekly and stored according to the manufacturer’s suggestions (21). Mice were treated twice a day at 12-h intervals via oral gavage for the duration of each study.

### Evaluation of Skeletal Muscle Function, Exercise Capacity, and Locomotor Behaviors

Male and female mice received either Veh or EZM for 11 days (*n* = 4 or 5 animals of each sex per group) or 13 days (*n* = 5 or 6 mice of each sex per group). On the 8th and 9th days of treatment, mice underwent a brief familiarization for each in vivo functional test (detailed in the following paragraphs). On the 10th day of treatment, functional assessments were completed on all mice. The animals treated for 11 days were euthanized on the 11th day ∼24 h after the cessation of the final test, whereas the cohort of remaining mice performed a single bout of treadmill running on the 11th day to assess exercise capacity, and their tissues were collected on the 13th day, ∼48 h after the treadmill run.

Mice underwent forelimb and all limb grip strength measurements (23) by pulling on a grid-grip dynamometer (Columbus Instruments; Columbus, OH). On the testing day, each mouse performed three successive pull attempts and was then returned to their cage for a rest period of 1 min. This was repeated five times, for a total of 15 attempts. Maximum grip strength was determined by taking the average of the three highest successive values out of the 15 pulls recorded and normalizing to body weight in grams. Fatigue was determined by calculating the decrement between the average of the first two series of attempts (1 + 2 + 3 = A, 4 + 5 + 6 = B) and the last two series of pulls (10 + 11 + 12 = C, 13 + 14 + 15 = D) using formula (C + D)/(A + B).

Rotarod testing was performed to evaluate motor coordination (23). During data collection, the mouse was placed on the rotarod, which accelerated from 5 to 45 rpm over 300 s, followed by an additional 300 s at 45 rpm. The time and speed at failure were recorded. The test was performed three times for each animal, and the best of these trials was used for statistical analysis.

The hang test examined mice for balance and muscular endurance (23). For this test, mice were placed on a metal cage lid and once the mouse grasped the lid, it was inverted and held in the same position for the duration of the test. The time at failure was recorded. The mice performed three trials, and the best time (i.e., longest duration before falling) of these three trials was used for statistical analysis. If a mouse fell off within 3 s of hanging from the cage, the test was restarted, and that trial was not counted.

Movement behavior was measured using an open-field Opto-Varimex-5 Auto-Track (Columbus Instruments; Columbus, OH; 24). Mice were placed into the center of the open-field and underwent a 1-h data collection session in a quiet environment without disruption. A variety of activity measures were recorded, including distance traveled, average speed, and resting time.

For the assessment of exercise capacity, mice were challenged with a single bout of exhaustive exercise on a motor-driven rodent treadmill (Columbus Instruments; Columbus, OH). The exercise protocol began at 15 m/min and increased by 5 m/min at the 10- and 20-min marks. The incline began at 5° and increased by 5° at the 30-, 40-, and 70-min marks. If mice could continue running beyond 90 min, the speed was increased by 5 m/min every 5 min until exhaustion, which was determined by the cessation of exercise despite probing with a soft bristle brush for 5 s (25).

### Denervation Experiments

Male and female mice were randomized to receive either Veh or EZM for 11 days (*n* = 8 animals of each sex per group). On the 5th day of treatment, unilateral sectioning of the sciatic nerve was performed. This model of neurogenic muscle atrophy evokes a rapid and robust remodeling of skeletal muscle in the DEN limb, while also allowing for the use of the contralateral, innervated limb to serve as an intra-animal control (CON). Mice were subjected to 7 days of DEN, and on the 11th day, animals were euthanatized, and tissues were weighed and collected.

Surgery was performed as previously described (15, 17). Briefly, mice were anesthetized by isoflurane inhalation (Fresenius Kabi; Bad Homburg, HE, Germany) before surgery and continued to receive isoflurane via nose cone for the duration of the surgery. Before the operation, a 5 mg/kg subcutaneous injection of carprofen (Zoetis; Lincoln, NE) was given subcutaneously for postoperative analgesia. A 1–2 cm skin incision was made in the posterior thigh musculature, and blunt dissection was used to expose the sciatic nerve. Unilateral DEN of the lower limb was induced by excising a ∼0.5 cm section of the sciatic nerve in the right hind limb. The overlying musculature was sutured with silk (Ethicon Inc.; Somerville, NJ), and the skin was secured using veterinary staples (Mikron Precision Inc.; Gardena, CA). The mice recovered in their cage on a heating pad and were weighed and monitored daily.

### Protein Extraction and Quantification

A portion of frozen GAST muscle (∼30 mg) was mechanically crushed with a tissue pulverizer (Cellcrusher; Cork, Ireland) in a liquid nitrogen bath and placed in RIPA buffer (Sigma Aldrich; 20 mL of radioimmunoprecipitation assay (RIPA) buffer per 1 mg muscle weight) supplemented with a protease and phosphatase inhibitor cocktail (Roche; Laval, QC, Canada). One stainless steel ball was added into each sample tube and then loaded into a precooled Tissue Lyser (Qiagen; Toronto, ON, Canada) and run for 5 bouts of 30 s at a frequency of 20.0 1/s. The ball was then removed using clean tweezers, and samples were sonicated for 5 × 5 s at maximum power. Following this, samples were spun in a centrifuge at 14,000 *g* for 10 min at 4°C. The resulting supernates were then collected, and a bicinchoninic assay (BCA; Thermofisher Scientific, Toronto, ON, Canada) was performed to determine protein concentration. All samples were diluted with 4× loading buffer and ultrapure water to a final concentration of 2 μg/μL.

### Western Blotting

Samples (20 μg) were separated on a 4%–20% Criterion TGX precast protein gel (Bio-Rad Laboratories; Mississauga, ON, Canada) for 55 min at 200 V. Afterwards, proteins were transferred onto nitrocellulose membranes using a Trans-Blot Turbo transfer system (Bio-Rad Laboratories). Subsequently, a Stain-Free image of the membrane was obtained using ChemiDoc MP Imaging System (Bio-Rad Laboratories) to verify equal sample loading. Membranes were placed in blocking solution [5% BSA in 1× Tris-buffered saline with 1% Tween-20 (TBST)] for 1 h at room temperature, then incubated overnight at 4°C with primary antibodies. Blots were then washed in 1× TBST for 3 × 5 min and incubated in the appropriate secondary antibody (1:10,000) for 75 min at room temperature. Next, membranes were washed in 1× TBST buffer for 3 × 5 min again before applying luminol-based enhanced chemiluminescence reagent (Bio-Rad Laboratories) for visualization. Finally, membranes were imaged using ChemiDoc MP Imaging System, and densitometry was performed on Image Lab software (Bio-Rad Laboratories).

The following primary antibodies were used: CARM1 (1:5,000; A300-421A; Bethyl Laboratories; Montgomery, TX), ADMA-marked CARM1 substrates [CARM1 substrates; 1:1,000; a kind gift from Dr. Mark Bedford, MD Anderson Cancer Center, University of Texas (26)], ADMA-marked SWItch/Sucrose Non-Fermentable (SWI/SNF) complex subunit (*m-BAF155*^Arg1064^; 1:1,000; 94962; Cell Signaling, Danvers, MA), AMDA-marked polyadenylate-binding protein 1 (*m-PABP1*^Arg455/460^; 1:1,000; 3505S; Cell Signaling), PABP1 (1:1,000; 4992S; Cell Signaling), complexes I-V of mitochondrial oxidative phosphorylation (OXPHOS; 1:1,000; ab110413; Abcam, Cambridge, UK), muscle RING-finger protein-1 (MuRF1; 1:200; AF5366; 140 R&D Systems, Minneapolis, MN), muscle atrophy F-box (MAFbx; 1:1,000; AP2041; ECM Biosciences, Versailles, KY), ubiquitin-binding protein 62 (p62; 1:1,000; P0067; Sigma Aldrich), BCL2 interacting protein 3 (BNIP3; 1:1,000; 3769S; Cell Signaling), microtubule-associated protein 1A/1B-light chain 3 (LC3; 1:1,000; 4108S; Cell Signaling), phosphorylated 4E-binding protein-1 (*p-4EBP1*^Thr37/46^; 1:1,000; 2855S; Cell Signaling), 4EBP1 (1:1,000; 9452S; Cell Signaling), p-ribosomal protein s6 (p-*s6*^Ser235/236^; 1:1,000; 2211S; Cell Signaling), and s6 (1:1,000; 2217S; Cell Signaling). We attempted to measure the total BAF155 protein content, however, we were unsuccessful despite troubleshooting with different antibody concentrations and dilution media.

### RNA Isolation and Real-Time Polymerase Chain Reaction

RNA was isolated from frozen, powdered GAST muscle. All samples were homogenized in 1 mL of Trizol reagent (Invitrogen; Carlsbad, CA) using stainless steel lysing beads and placed in the Tissue Lyser (Qiagen) to run for 5 bouts of 30 s at a frequency of 20.0 1/s. Homogenized samples were then mixed with 200 mL of chloroform (Thermo Fisher Scientific; Waltham, MA), agitated vigorously for 15 s, and centrifuged at 12,000 *g* for 10 min. The upper aqueous (RNA) phase was purified using the E.Z.N.A total RNA kit (VWR International; Radnor, PA), as per the instructions provided by the manufacturer. RNA concentration and purity were determined using the NanoDrop 1000 Spectrophotometer (Thermo Fisher Scientific). RNA samples were then reverse transcribed into cDNA using a high-capacity cDNA reverse transcription kit (Thermo Fisher Scientific), according to the manufacturer’s instructions. All individual real-time polymerase chain reactions (RT-qPCRs) were run in triplicate 6 µL reactions containing GoTaq qPCR Master Mix (Promega; Madison, WI). Data were analyzed using the comparative threshold cycle (CT) method (27). TATA box binding protein (TBP) and GAPDH were used as control housekeeping genes for all experiments, as the average of their CT values did not change between experimental conditions (data not shown). The following primers (Sigma Aldrich) were used in this study: *Carm1* F-CAACAGCGTCCTCATCCAGT, R-GTCCGCTCACTGAACACAGA; fibroblast growth factor-binding protein (*Fgfbp1*) F-ACACTCACAGAAAGGTGTCCA, R-CTGAGAACGCCTGAGTAGCC; *Ppargc1α* F-AGTGGTGTAGCGACCAAT, R-GGGCAATCCGTCTTCATCCA. Notably, total *Ppargc1α* mRNA content was measured, irrespective of isoform.

### Immunofluorescence Microscopy

SOL muscles stored in OCT were sectioned into 10 mm slices on a cryostat (Thermo Fisher Scientific) at −20°C. Before staining, samples were air-dried for ∼30 min and subsequently incubated in 10% goat serum in PBS to prevent nonspecific binding. Staining of myosin heavy chain (MHC) isoforms was performed, as previously described (28), using primary antibodies against MHC I (BA-F8), MHC IIa (SC-71), and MHC IIb (BF-F3; Developmental Studies Hybridoma Bank, Iowa City, IA), followed by isotype-specific fluorescent secondary antibodies (Invitrogen, Carlsbad, CA). This allowed for the identification of type I, type IIa, type IIb, type IIx, and hybrid muscle fibers. All slides were viewed with the Nikon Eclipse Ti Microscope (Nikon Instruments, Mississauga, ON, Canada), equipped with a high-resolution Photometrics CoolSNAP HQ2 fluorescent camera. Images were captured and analyzed using the Nikon NIS Elements AR 3.2 software. All images were obtained with the ×20 objective. For each sample, all fibers of every type were counted to obtain fiber type percentage. The fibers within a predetermined region of interest (which excluded exterior and elongated fibers) were manually circled to determine fiber-type specific cross-sectional area (CSA). Investigators performing counting and circling were blinded to the experimental conditions.

### Statistical Analyses

**Figure 1.**
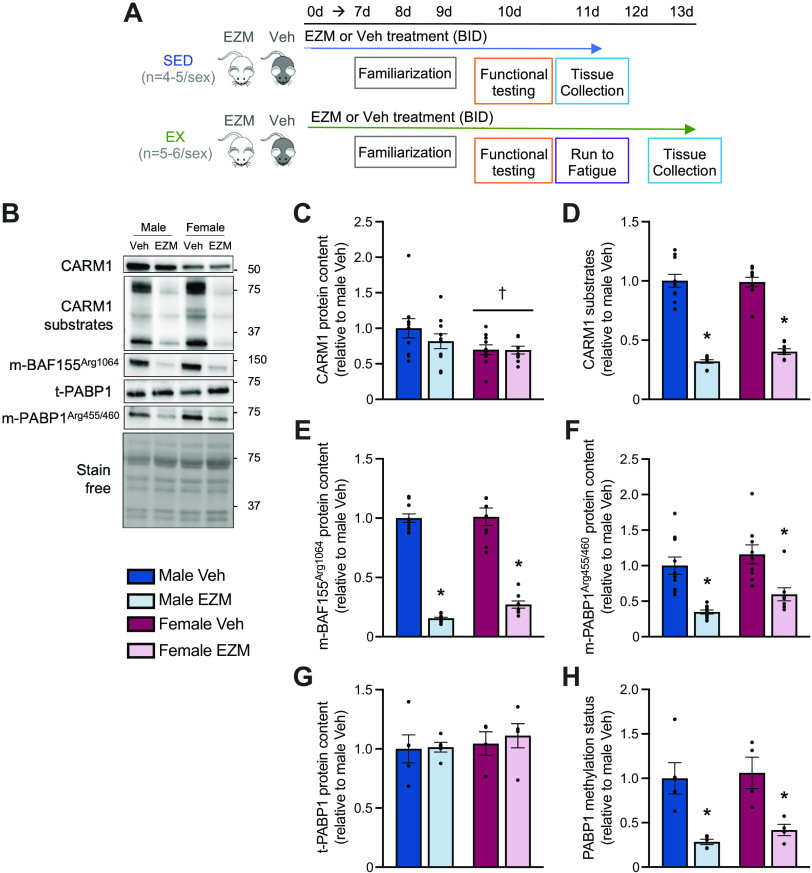
EZM inhibits CARM1 in skeletal muscle. *A*: experimental design for the first arm of this study. *B*: typical Western blots of CARM1, CARM1 substrates, arginine methylated (m)-*BAF155*^Arg1064^, *m-PABP1*^Arg455/460^, and total (t)-PABP1 protein in GAST muscles of male and female mice after 11–13 days of daily Veh or EZM (150 mg/kg) treatment. A stain free image of the membrane is shown to demonstrate equal sample loading. Approximate molecular weights (kDa) are displayed at right of blots. Graphical summaries of CARM1 protein (*C*), CARM1 substrates (*D*), *m-BAF155*^Arg1064^ (*E*), *m-PABP1*^Arg455/460^ (*F*), t-PABP1 (*G*), and PABP1 methylation status (i.e., the methylated form of the protein relative to its total amount; *H*), expressed relative to the male Veh-treated group. Bar graphs are expressed as means ± SE, and individual data points in each group are shown. Two-way ANOVA; *P < 0.05 vs. Veh-treated group of same sex; †main effect of sex. *n* = 5–11. CARM1, coactivator-associated arginine methyltransferase 1; EZM, EZM2302; GAST, gastrocnemius; Veh, vehicle.

**Figure 2.**
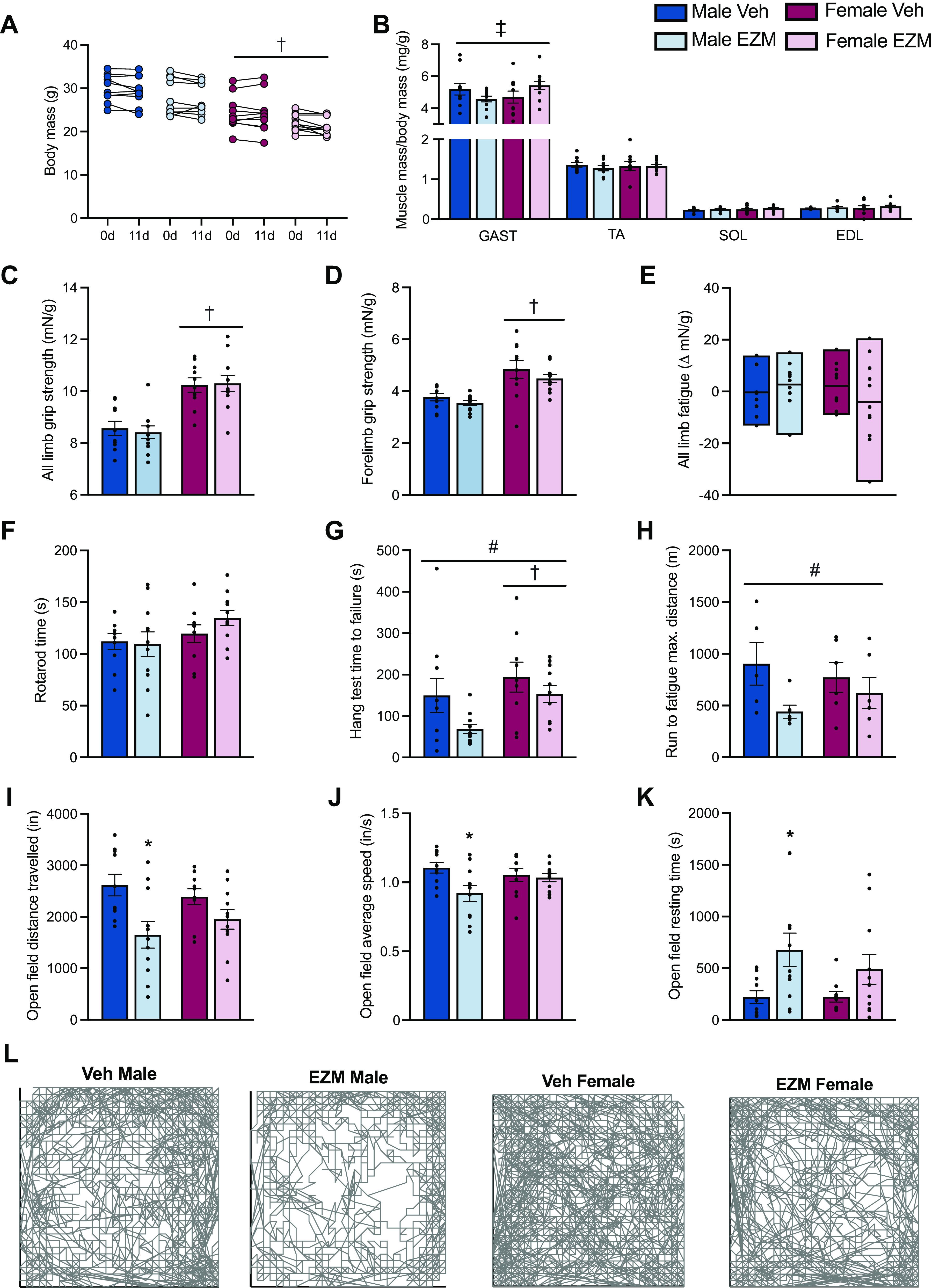
Daily CARM1 inhibition impacts skeletal muscle function, exercise capacity, and movement behaviors. *A*: body mass of male and female mice at 0 and 11 days of treatment with EZM or Veh compound. *B*: mass of gastrocnemius (GAST), tibialis anterior (TA), soleus (SOL), and extensor digitorum longus (EDL) muscles expressed relative to body mass (mg/g) of male and female EZM and Veh-treated mice. Maximum all limb (*C*) and forelimb grip (*D*) strength expressed relative to body weight (mN/g). *E*: all-limb grip fatigue (ΔmN/g). *F*: maximum run time (s) during rotarod test. *G*: time to failure (seconds; s) during hang test. *H*: maximum distance traveled (meters; m) during treadmill exercise. *I–K*: distance traveled (inches; in.), average speed (inches/second; in./s), and resting time (s) in the four experimental groups during a 60-min open field tracking period. *L*: representative tracings of movement patterns during open field test. Bars are means ± SE, and individual data points in each group are shown. Two-way ANOVA, *P < 0.05 vs. Veh-treated group of same sex: †main effect of sex; ‡sex and treatment interaction; #main effect of treatment. *n* = 10 or 11 in *A–K*. *n* = 5 or 6 in *H*. CARM1, coactivator-associated arginine methyltransferase 1; EZM, EZM2302; Veh, vehicle.

**Figure 3.**
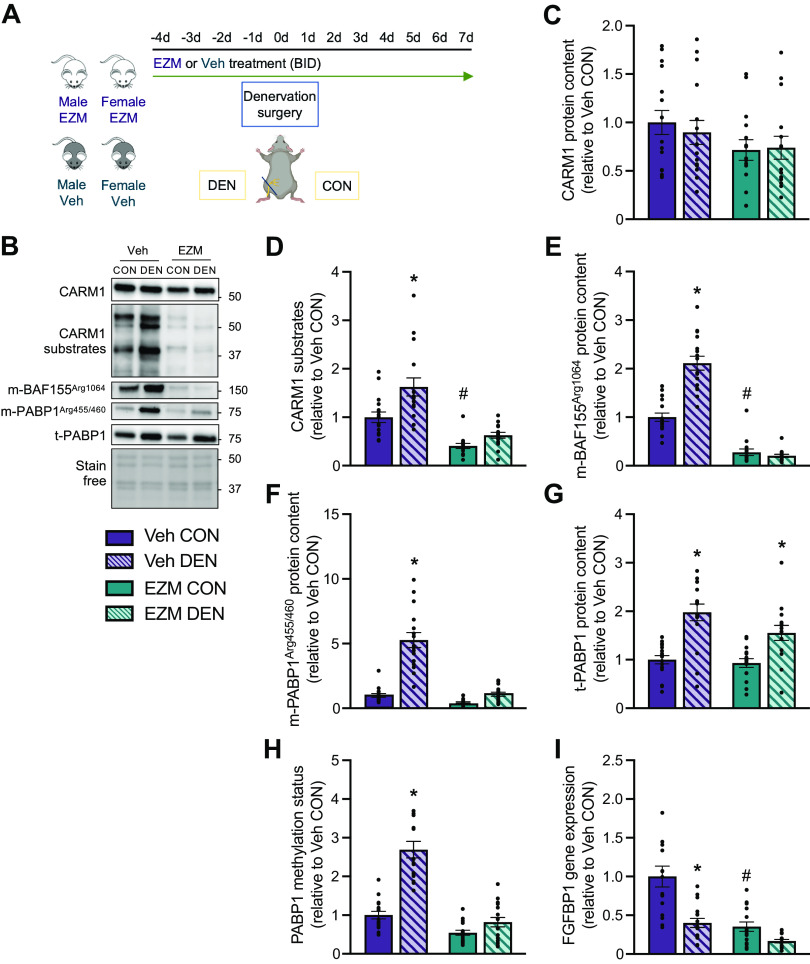
Pharmacological CARM1 inhibition attenuates increases in CARM1 methyltransferase activity following 7 days of denervation-induced neurogenic disuse. *A*: experimental design for the second arm of this study. *B*: representative Western blots of CARM1, CARM1 substrates, *m-BAF155*^Arg1064^, *m-PABP1*^Arg455/460^, and t-PABP1 in GAST muscles of CON and DEN limbs of Veh- and EZM-treated mice. A stain-free image of the membrane is shown to demonstrate consistent loading. Approximate molecular weights (kDa) are displayed at right of blots. Graphical summaries of CARM1 (*C*), CARM1 substrates (*D*), *m-BAF155* ^Arg1064^ (*E*), *m-PABP1*^Arg455/460^ (*F*), t-PABP1 (*G*), and PABP1 methylation status (*H*) expressed relative to Veh CON. *I*: graphical summary of *FGFBP1* mRNA expression in GAST muscles shown relative to Veh CON. Male and female data were pooled because there was no significant difference between sexes in the percentage change in these outcome measures in response to DEN (data not shown). Bar graphs are expressed as means ± SE, and individual data points for each group are shown. Two-way ANOVA; *P < 0.05 between DEN and CON groups; #*P* < 0.05 between Veh CON and EZM CON groups. *n* = 15 or 16. CARM1, coactivator-associated arginine methyltransferase 1; CON, control; DEN, denervation; EZM, EZM2302; GAST, gastrocnemius; Veh, vehicle.

**Figure 4.**
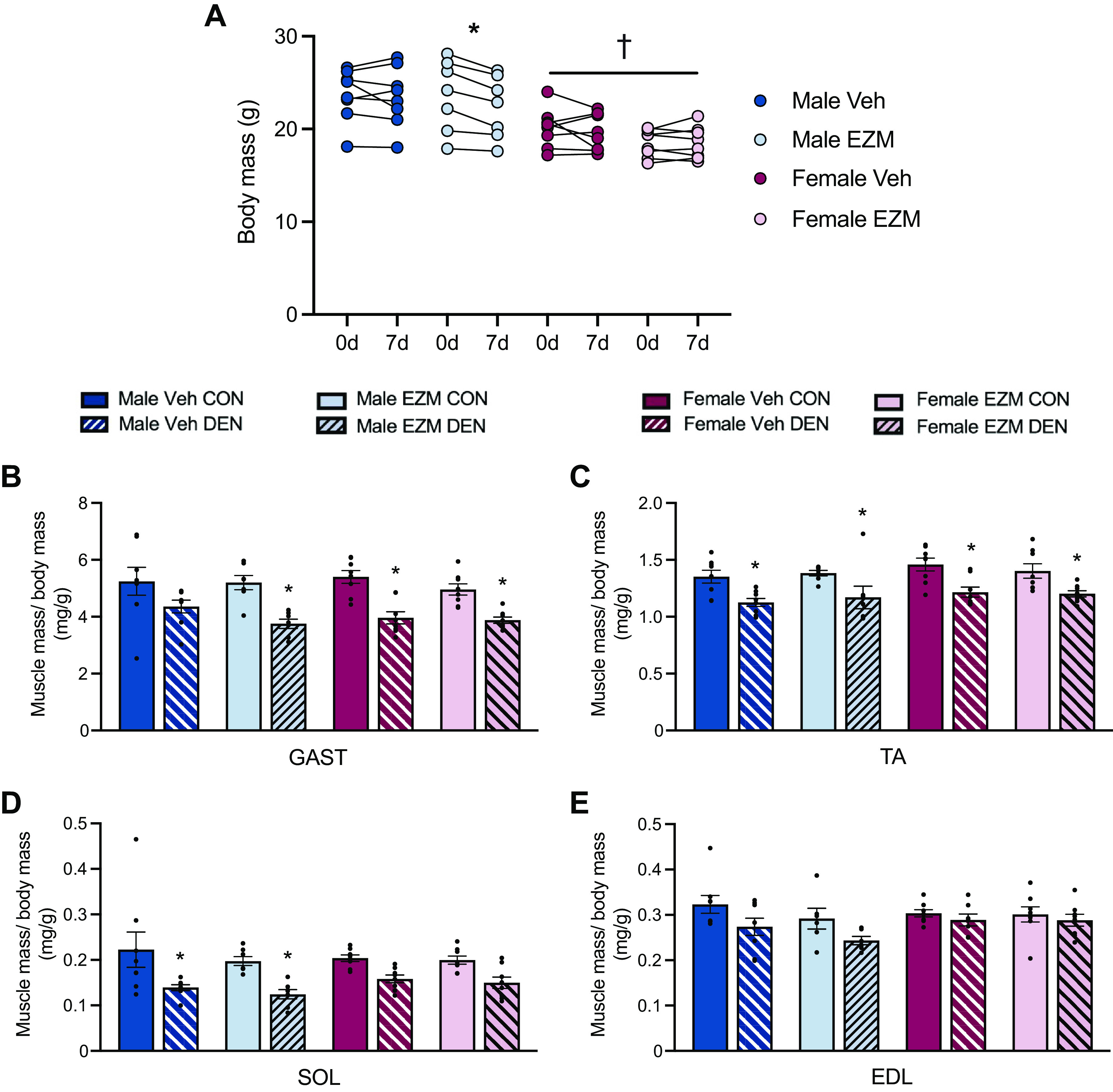
CARM1 inhibition does not influence denervation-induced muscle mass loss. *A*: graphical summary of body mass (g) before DEN surgery (*0 day*) and after surgery (*7 days*) in male and female mice receiving EZM or Veh. Summaries of muscle mass expressed relative to body mass (mg/g) in GAST (*B*), TA (*C*), SOL (*D*), and EDL (*E*) in the CON and DEN limbs of mice across the four experimental groups. Bar graphs are means ± SE, and individual data points for each group are shown. Three-way ANOVA; *P < 0.05 between DEN and CON groups; †main effect of sex. *n* = 7 or 8. CARM1, coactivator-associated arginine methyltransferase 1; CON, control; DEN, denervation; EDL extensor digitorum longus; EZM, EZM2302; GAST, gastrocnemius; SOL, soleus; TA, tibialis anterior; Veh, vehicle.

**Figure 5.**
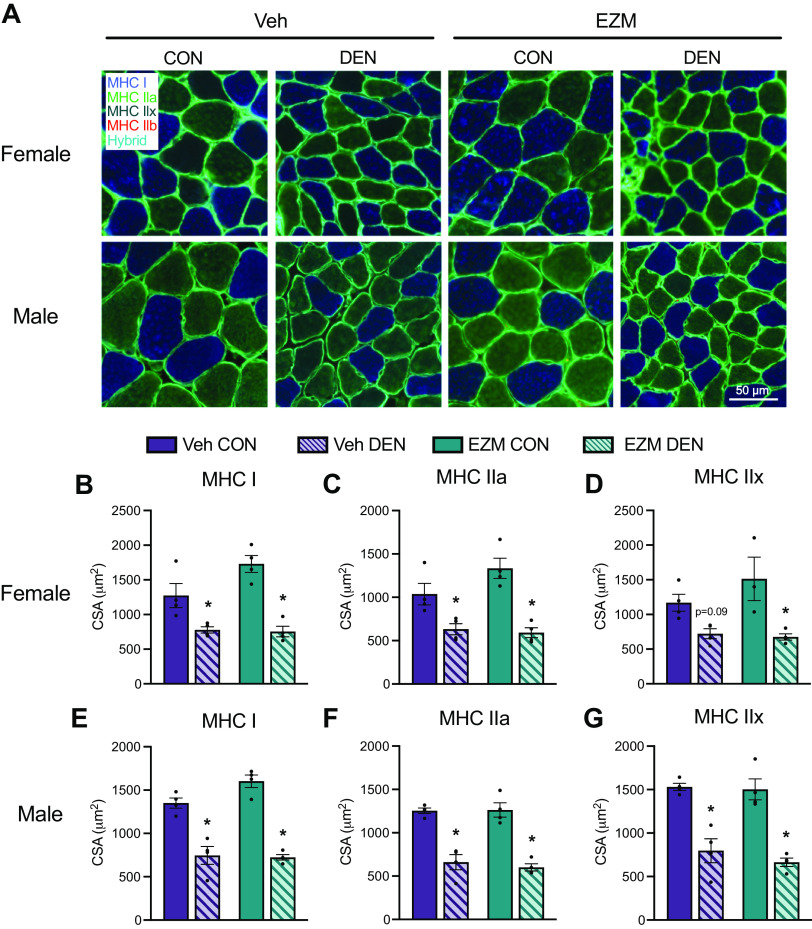
Denervation decreases muscle fiber cross-sectional area independent of pharmacological CARM1 inhibition. *A*: representative images of immunofluorescence fiber-type staining of soleus muscles, including laminin (cyan), myosin heavy chain (MHC) type I (blue), MHC type IIa (green), and MHC IIb (red). Type IIx fibers were unstained, and type I/IIa hybrid fibers appear as a blue/green hybrid. Graphical summary of average cross-sectional area (CSA; μm^2^) of MHC I (*B*), MHC IIa (*C*), and MHC IIx (*D*) fibers of DEN and CON muscle sections from female EZM and Veh-treated mice. Graphical summary of average CSA of MHC I (*E*), MHC IIa (*F*), and MHC IIx-stained (*G*) fibers of DEN and CON muscle sections from male EZM and Veh-treated mice. Data are expressed as means ± SE. Two-way ANOVA; *P < 0.05 between DEN and CON groups. *n* = 4. CARM1, coactivator-associated arginine methyltransferase 1; CON, control; DEN, denervation; EZM, EZM2302; Veh, vehicle.

**Figure 6.**
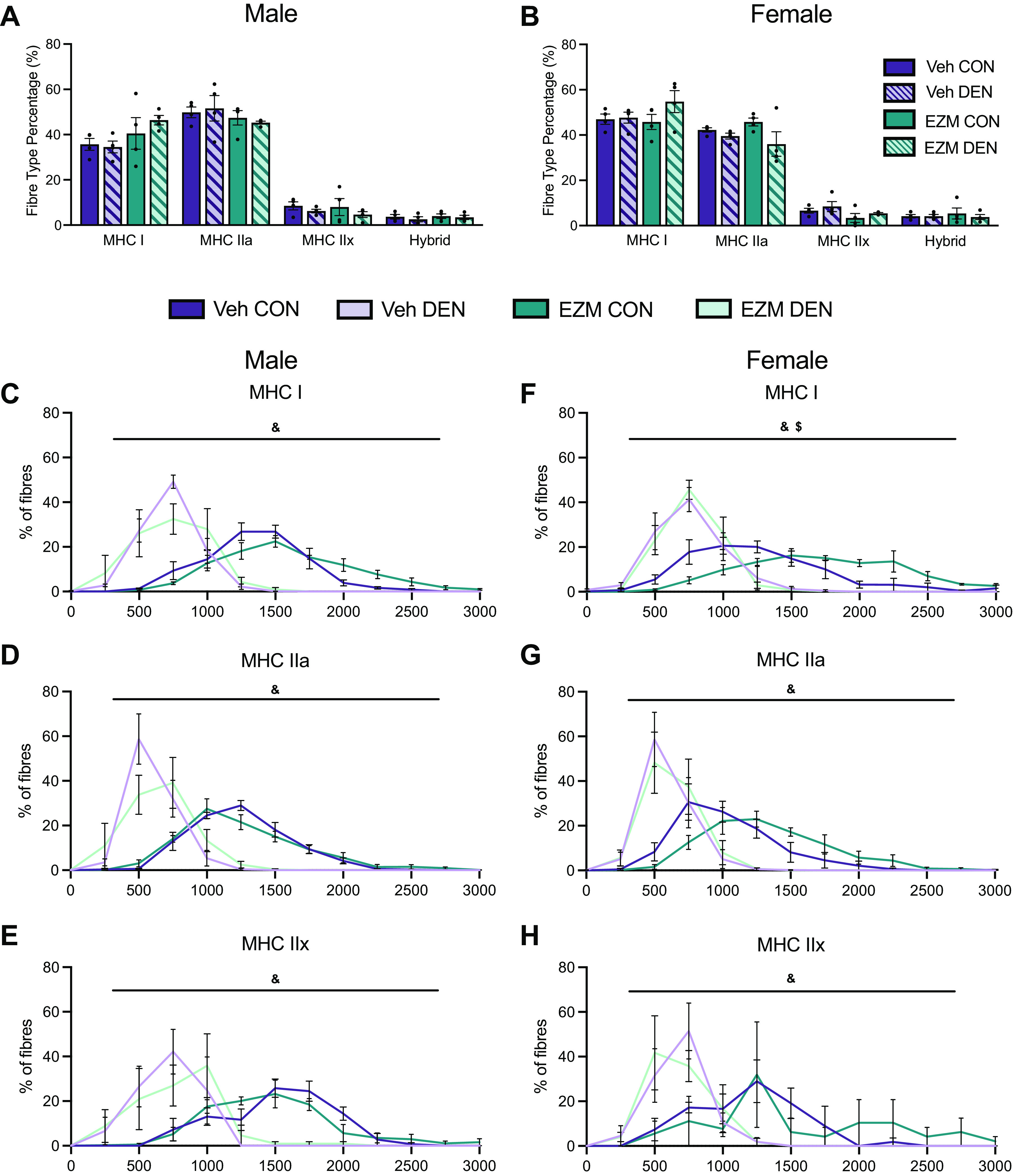
CARM1 inhibition does not influence fiber type composition distribution. Bar graphs of fiber type distribution in CON and DEN SOL muscles of male (*A*) and female (*B*) EZM and Veh-treated mice. *C–E*: fiber size distribution of type I, type IIa, and type IIx fibers in CON and DEN muscles of male mice. *F–H*: fiber size distribution of type I, type IIa, and type IIx fibers in CON and DEN muscles of female mice. Data are expressed as means ± SE. Two-way ANOVA for *A* and *B*; three-way ANOVA for *C–H*; &size and DEN interaction, $size and drug interaction. *n* = 4. CARM1, coactivator-associated arginine methyltransferase 1; CON, control; DEN, denervation; EZM, EZM2302; SOL, soleus; Veh, vehicle.

**Figure 7.**
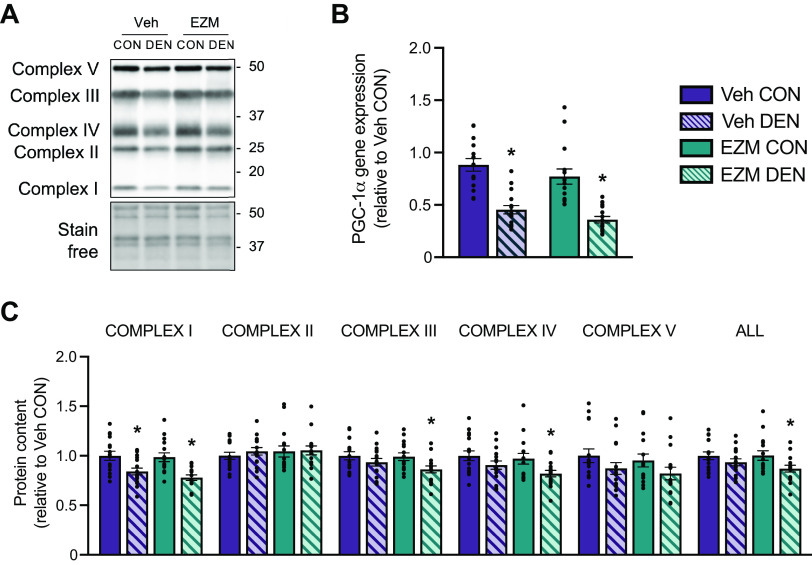
CARM1 inhibition affects the mitochondrial protein profile in response to denervation. *A*: typical Western blots of OXPHOS complexes I-V in GAST muscles from CON and DEN muscles of Veh and EZM-treated mice. A stain-free image of the membrane is shown to demonstrate consistent loading. Approximate molecular weights (kDa) are displayed at right of blots. *B*: summary of *PGC-1α* mRNA expression in GAST muscles shown relative to Veh CON. *C*: graphical summary of mitochondrial complex I-V protein levels in CON and DEN limbs of EZM and Veh-treated mice. Data are expressed relative to the Veh CON condition. Male and female data were pooled because there was no significant difference between sexes in the percentage change in these outcome measures in response to DEN (data not shown). Bar graphs are means ± SE. Two-way ANOVA; *P < 0.05 between DEN and CON groups. *n* = 15 or 16. CARM1, coactivator-associated arginine methyltransferase 1; CON, control; DEN, denervation; EZM, EZM2302; GAST, gastrocnemius; OXPHOS, mitochondrial oxidative phosphorylation; PGC-1α, peroxisome proliferator-activated receptor-γ coactivator-1α; SOL, soleus; Veh, vehicle.

**Figure 8.**
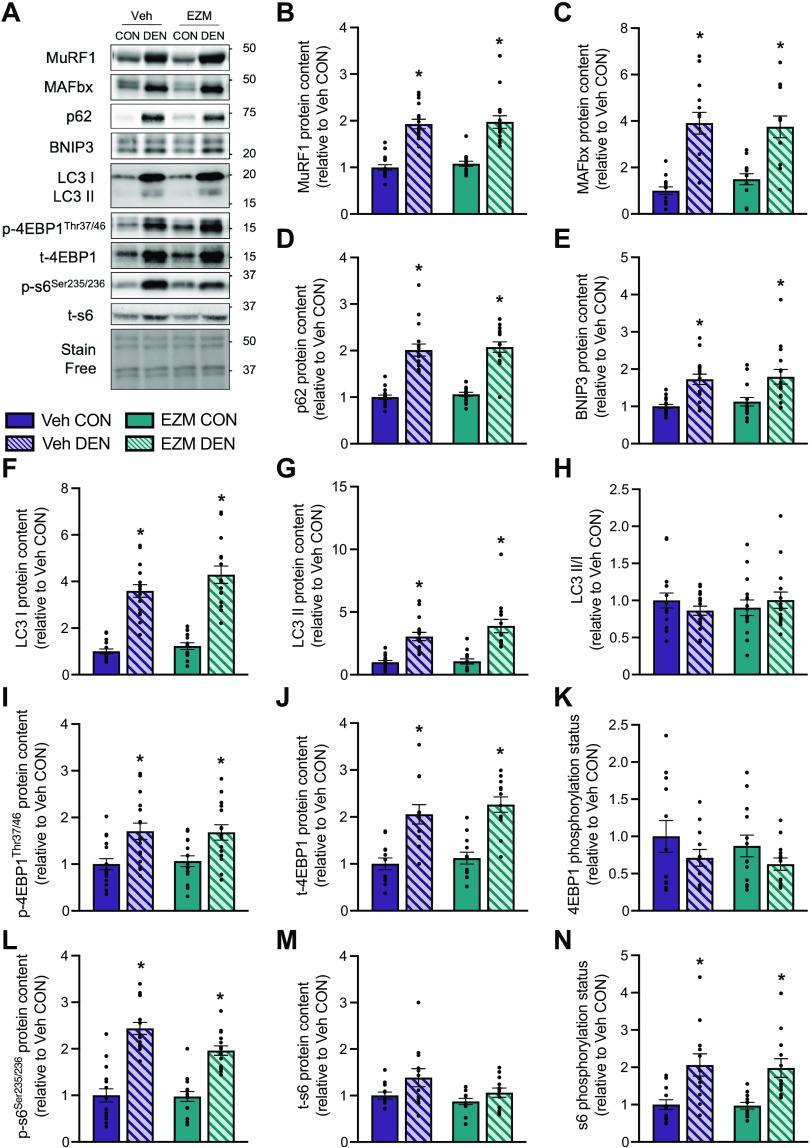
Molecular regulators of skeletal muscle size are elevated following denervation independent of pharmacological CARM1 inhibition. *A*: representative Western blots of MURF1, MAFbx, p62, BNIP3, LC3 I/II, phosphorylated (p)-*4EBP1*^Thr37/46^, t-4EBP1, *p-s6*^Ser235/236^, and t-s6 in CON and DEN limbs of Veh- and EZM-treated mice. A representative stain free image of the membrane is shown below to demonstrate equal sample loading. Approximate molecular weights (kDa) are displayed at right of blots. Graphical summaries of MURF1 (*B*), MAFbx (*C*), p62 (*D*), BNIP3 (*E*), LC3 I (*F*), LC3 II (*G*), LC3 II/I ratio (*H*), *p-4EBP1*^Thr37/46^ (*I*), t-4EBP1 (*J*), 4EBP1 phosphorylation status (i.e., the phosphorylated form of the protein relative to its total amount; *K*), *p-s6*^Ser235/236^ (*L*), t-s6 (*M*), and s6 phosphorylation status (*N*) in CON and DEN limbs of both treatment groups expressed relative to the Veh CON cohort. Male and female data were pooled because there was no significant difference between sexes in the percentage change in these outcome measures in response to DEN (data not shown). Data are expressed as means ± SE. Two-way ANOVA; *P < 0.05 between DEN and CON groups. *n* = 12–16. CARM1, coactivator-associated arginine methyltransferase 1; CON, control; DEN, denervation; EZM, EZM2302; Veh, vehicle.

All statistical analyses were completed on Prism. A two-way analysis of variance (ANOVA) was performed to examine the main effects of treatment (EZM vs. Veh) and sex (male vs. female), as well as any interactions between variables, for protein content, muscle mass, and functional measures in Figs. 1 and 2. In Figs. 3, 7, and 8, when there was no difference in percentage change in protein or mRNA content between male and female mice in response to DEN, male and female data were pooled to increase our statistical power to detect the true effects of experimental conditions. Subsequently, a two-way ANOVA was completed to investigate the main effects of treatment and DEN, as well as interactions between variables. A three-way ANOVA was performed to examine the main effects of treatment, sex, and DEN (CON vs. DEN) and interactions between variables, for body and muscle mass in Fig. 4. In Figs. 5 and 6, a two-way ANOVA was completed separately in male and female mice to assess main effects of DEN and treatment, as well as interactions between variables. Šidák multiple comparisons tests were performed when main effects and/or interactions were identified. Statistical differences were considered significant if *P* < 0.05. Data are presented as means ± SE.

## RESULTS

### Pharmacological CARM1 Inhibition Reduces CARM1 Methyltransferase Activity In Skeletal Muscle

To examine the effects of global, pharmacological CARM1 inhibition on skeletal muscle biology, adult male and female mice (*n* = 10 or 11/sex) were treated with either EZM (150 mg/kg) or Veh compound for 11–13 days (Fig. 1*A*). On the 10th day of treatment, a variety of functional tests were completed on all mice. The animals treated for 11 days were euthanized on *day 11*, whereas the cohort of remaining mice performed a single bout of treadmill running on *day 11* to assess exercise capacity, and their tissues were collected on *day 13*. We sought to investigate CARM1 biology in muscles following a longer duration of EZM exposure. We performed immunoblot analyses of CARM1, and its known targets, including PABP1, BAF155, and CARM1 substrates in the GAST muscles of Veh- and EZM-treated male and female animals. We observed a main effect of sex, where skeletal muscle CARM1 content was 25% lower in female versus male mice (Fig. 1, *B* and *C*). CARM1 content was unaffected by EZM administration. ADMA-marked CARM1 substrates and methylated *BAF155*^Arg1064^ levels were decreased (*P* < 0.05) by 65%–80% in EZM-treated male and female mice compared with Veh-treated animals (Fig. 1, *B*, *D*, and *E*). EZM significantly reduced asymmetrically dimethylated *PABP1*^Arg455/460^ content and PABP1 methylation status (i.e., the methylated form of the protein expressed relative to the total protein content) by 50%–75% in both sexes versus Veh-dosed mice (Fig. 1, *B* and *F–H*).

### Pharmacological Inhibition of CARM1 Does Not Affect Body or Skeletal Muscle Mass but Impairs Muscle Function in a Sex-Specific Manner

Next, we analyzed body mass in response to EZM administration and found that it was unchanged after 11 days of treatment (Fig. 2*A*). EZM-treated male mice had significantly reduced GAST muscle mass, as compared with Veh-treated males, whereas female mice treated with EZM demonstrated a greater (*P* < 0.05) GAST mass versus their sex-matched Veh counterparts (Fig. 2*B*). However, the mass of several other lower limb muscles was not affected by EZM. Grip strength and grip fatigue were similar between EZM- and Veh-treated mice (Fig. 2, *C*–*E*).

Muscular endurance and coordination were assessed via the hang and rotarod tests (23, 29). Rotarod performance was similar across treatment groups and sex (Fig. 2*F*). In contrast, we observed the main effects of treatment and sex in the time to failure during the hang test, where EZM-treated female and male mice fatigued 40%–55% earlier than Veh-treated animals (Fig. 2*G*). Male EZM-treated mice tended to run less (*P* = 0.08) than Veh-treated male mice during the treadmill test, whereas exercise capacity was similar between female Veh and EZM groups (Fig. 2*H*). We next examined the effects of pharmacological CARM1 inhibition on locomotor and exploratory behaviors by monitoring the activity of mice during a 1-h tracking period in an open-field chamber (30). EZM-dosed male mice demonstrated significantly lower movement speed, less distance traveled, and more sedentary time compared with their sex-matched Veh-treated littermates (Fig. 2, *I–L*). In contrast, there was no difference in open-field parameters between treatment groups in female mice.

### Pharmacological CARM1 Inhibition Attenuates Increased CARM1 Methyltransferase Activity Following Denervation

We sought to explore the impact of pharmacological CARM1 inhibition on denervation-induced muscle atrophy. Our pilot experiments demonstrated that 4 days of EZM administration was effective at significantly decreasing asymmetric dimethylation of arginine residues on CARM1 substrates by 85% in liver and 70% in skeletal muscle (data not shown). Therefore, female and male mice were pretreated for 4 days with either EZM or Veh compounds, and on *day 5* of treatment, unilateral sectioning of the sciatic nerve was performed (15, 17) to rapidly evoke neurogenic muscle atrophy in one limb, while maintaining a contralateral, innervated intra-animal control limb (Fig. 3*A*). The Veh/EZM course continued for another 7 days, and animals were euthanized on *day 11*. To investigate whether CARM1 inhibition was effective at mitigating elevations in CARM1 methyltransferase activity following DEN, we immunoblotted CARM1 and its substrates in control (CON) and denervated (DEN) GAST muscles of Veh- and EZM-treated male and female mice. To increase our statistical power to detect the effects of EZM treatment and DEN, male and female data were pooled because there were no significant differences between sexes in the percentage change of these outcome metrics in response to DEN (data not shown). CARM1 protein content was unaffected by DEN in either treatment group, although it tended to be reduced (*P* = 0.07) in EZM-dosed mice (Fig. 3, *B* and *C*). ADMA-marked CARM1 substrates and *BAF155*^Arg1064^ were significantly elevated 1.6- and twofold, respectively, in the DEN muscles of Veh-treated but not in EZM-treated mice (Fig. 3, *B*, *D*, and *E*). Similarly, ADMA-marked *PABP1*^Arg455/460^ was increased (*P* < 0.05) fivefold in DEN GAST muscle compared with CON in Veh-dosed mice, however, this induction was significantly attenuated in EZM-dosed mice (Fig. 3, *B* and *F*). Total PABP1 levels were 1.6–2-fold greater (*P* < 0.05) in DEN versus CON muscles of both groups, with no difference between Veh and EZM administration (Fig. 3, *B* and *G*). EZM significantly blunted the elevation in PABP1 methylation status observed in DEN muscles of Veh-treated mice (Fig. 3*H*). vanLieshout et al. (8) recently identified several genes that were differentially expressed in the skeletal muscles of CARM1 mKO mice compared with their wild-type (WT) littermates, indicating that these transcripts were regulated by CARM1. Specifically, mRNA levels of *FGFBP1* were reduced by ∼95% (*P* < 0.05) in CARM1 mKO mice. Similarly, here we found that *FGFBP1* mRNA expression was significantly 65% lower in the CON muscle of EZM-treated mice compared with their Veh-treated counterparts and was decreased (*P* < 0.05) following DEN in both treatment groups (Fig. 3*I*).

### Denervation-Induced Muscle Mass Loss is Unaffected by Pharmacological CARM1 Inhibition

We investigated the effect of global, pharmacological CARM1 inhibition on body and skeletal muscle mass in response to DEN-induced muscle atrophy. We found that EZM-dosed male mice had significantly lower body mass post-DEN versus pre-DEN, whereas the other treatment groups exhibited no change in body mass in response to neurogenic disuse (Fig. 4*A*). There was a 15%–20% reduction (*P* < 0.05) in DEN GAST muscle mass, expressed relative to body mass, compared with its innervated, contralateral CON across all groups except the male Veh cohort, which demonstrated a strong statistical trend (*P* = 0.06; Fig. 4*B*). TA muscle mass was significantly reduced by ∼15% in the DEN versus CON limbs in Veh- and EZM-treated mice of both sexes (Fig. 4*C*). DEN significantly decreased SOL muscle mass in all male mice, however, a statistical trend (*P* < 0.13) for SOL mass reduction in DEN compared with CON legs was observed in female animals (Fig. 4*D*). EDL muscle mass was not affected by neurogenic disuse (Fig. 4*E*).

### Denervation Reduces Muscle Fiber Size Independent of Pharmacological CARM1 Inhibition

Next, we examined the influence of DEN on muscle fiber-specific CSA and fiber type composition distribution to determine whether these factors were affected by CARM1 inhibition. Following 7 days of DEN, SOL muscle fibers appear smaller and more angular than CON fibers (Fig. 5*A*). In female mice, MHC type I and IIa fiber types exhibited a significant decrease in CSA after DEN that was independent of CARM1 inhibition (Fig. 5, *A*–*C*). A similar effect was observed in MHC type IIx fibers where a statistical trend (*P* < 0.09) was demonstrated (Fig. 5, *A* and *D*). Similarly, the CSA of myosin heavy chain (MHC) type I, IIa, and IIx DEN fibers in male mice was also reduced (*P* < 0.05) relative to CON fibers in both EZM- and Veh-treated groups (Fig. 5, *A* and *E–G*). The SOL muscles of female mice had the following distribution of MHC I, IIa, IIx, IIb, and hybrid fibers: 48%, 40%, 7%, 1%, and 4%, whereas the SOL muscle of male mice had the following distribution: 37%, 50%, 8%, 1%, and 4% (Fig. 6, *A* and *B*). There was no significant effect of DEN or EZM treatment on fiber type distribution in either sex. In male and female mice, there was an interaction between DEN and size of MHC I, IIa, and IIx fibers, such that a greater percentage of fibers in DEN muscle had smaller CSA than in CON muscle (Fig. 6, *C*–*H*). In type I fibers of female mice, EZM CON muscles tended to have more large fibers compared with Veh CON, an effect that was not apparent in the DEN muscles (Fig. 6*F*).

### Pharmacological CARM1 Inhibition Affects Mitochondrial Protein Profile in Response to Denervation

We then studied, during neurogenic atrophy, the effect of CARM1 inhibition on PGC-1α expression, a potent regulator of mitochondrial biogenesis in skeletal muscle. *PGC-1α* mRNA content was lower (*P* < 0.05) in DEN muscles of both EZM and Veh-treated mice relative to the CON condition (Fig. 7*B*). We also examined the expression of OXPHOS complexes I-V (CI-CV). We observed a significant ∼20% reduction in CI content in DEN muscles compared with CON in both Veh- and EZM-treated groups, however, CIII and CIV levels were only decreased (*P* < 0.05) in DEN muscles of EZM-dosed mice, whereas CII and CV were similar between all groups (Fig. 7, *A* and *C*). When the data for CI-CV were pooled, DEN lowered OXPHOS expression by 10%–15% in Veh- and EZM-treated mice, but the reduction was statistically significant only in the EZM groups (Fig. 7, *A* and *C*).

### Molecular Regulators of Skeletal Muscle Mass Are Elevated following Denervation Independently of Pharmacological CARM1 Inhibition

Finally, we investigated the effects of pharmacological CARM1 inhibition on several molecules that are well-characterized mediators of skeletal muscle mass. Muscle-specific ubiquitin ligases, MuRF1 and MAFbx, were significantly upregulated two- to fourfold, respectively, in DEN relative to CON muscles in both treatment groups, with no difference between Veh and EZM (Fig. 8, *A*–*C*). Proteins that are indicative of autophagy and mitophagy, p62 and BNIP3, were also elevated approximately twofold (*P* < 0.05) in DEN muscles and were unaffected by pharmacological CARM1 inhibition (Fig. 8, *A*, *D*, and *E*). We also examined LC3, an autophagosome membrane protein that is converted from LC3 I to LC3 II during the formation of autophagosomes (31), in response to DEN and EZM. We found that LC3 I and II were robustly increased (*P* < 0.05) in DEN muscles compared with CON to a similar extent between Veh and EZM groups, however, the LC3II-to-LC3-I ratio was unaffected (Fig. 8, *A* and *F–H*). Finally, we measured downstream targets of mammalian target of rapamycin (mTOR), s6 and 4EBP1, as molecular outputs of mTOR activity during neurogenic muscle atrophy. Phosphorylated *4EBP1*^Thr37/46^ and total 4EBP1 levels were significantly greater in DEN versus CON muscles with no difference between Veh and EZM administration, whereas 4EBP1 phosphorylation status was similar across all groups (Fig. 8, *A* and *I–K*). Phosphorylated *s6* ^Ser235/236^ and total s6 were increased more in DEN muscles of Veh-treated mice than EZM-treated mice, however s6 phosphorylation status was similar between treatment groups (Fig. 8, *A* and *L–N*).

## DISCUSSION

The purpose of the present study was to further investigate the role of CARM1 in the maintenance and plasticity of skeletal muscle mass and function at rest and during neurogenic muscle atrophy. Our data demonstrate that short-term treatment with an orally bioavailable, CARM1 inhibiting compound was effective at reducing CARM1 activity in skeletal muscle. The decrease in CARM1 methyltransferase function corresponded with impaired muscular endurance and exercise capacity, particularly in male mice, despite only minimal changes to body mass and muscle mass. DEN augmented the methylation of CARM1 substrates in skeletal muscle of Veh-treated mice, but this response was abolished in EZM-treated animals. Nevertheless, DEN-induced decreases in skeletal muscle mass and myofiber CSA occurred to a similar degree in males and females irrespective of CARM1 activity. Accordingly, the molecular muscle atrophy and autophagy programs were upregulated following DEN independent of CARM1 methylating capacity. Collectively, these data demonstrate that pharmacological CARM1 inhibition markedly reduced CARM1 activity in skeletal muscle while eliciting alterations in muscle and whole body physical function in the absence of changes in muscle mass and myofiber CSA. Furthermore, short-term CARM1 suppression had no impact on DEN-evoked muscle atrophy. These findings, along with other recent work (8, 15, 17, 18), together indicate specific spatiotemporal effects of CARM1 content and activity in the maintenance and remodeling of skeletal muscle biology.

We used a novel pharmacological strategy that targets CARM1 to further evaluate the role of the methyltransferase in skeletal muscle of adult animals. Although not without drawbacks such as broad, “off-target” tissue pharmacokinetics, this methodology circumvents the major limitations associated with common genetic approaches utilizing full-body or skeletal muscle-specific CARM1 KO (4, 5, 8, 15). Furthermore, the CARM1 inhibitor EZM is orally bioactive, highly specific, and safe in several preclinical contexts (21, 22, 32–34), and therefore represents an effective and practical tool compound with significant translational potential (20). EZM directly binds to CARM1 at the peptide-substrate binding site to prevent methylation but does not contribute to the degradation of the enzyme (34). As such, muscle CARM1 protein content was not different between Veh- and EZM-treated animals. On the other hand, EZM administration significantly decreased markers of skeletal muscle CARM1 activity, including specific *BAF155*^Arg1064^ and *PABP1*^Arg455/460^ methylation and general asymmetric dimethylation of CARM1 substrates, by 55%–80% compared with Veh-treated animals. In fact, this EZM-evoked reduction in GAST muscle CARM1 arginine methyltransferase activity was greater than that observed in the TA muscle of CARM1 mKO mice as compared with age- and sex-matched wild-type littermates [−40%–65% (8, 15)], which is likely due to the added inhibition by EZM of CARM1 in muscle-resident nonmyogenic cells. We also show that global CARM1 inhibition under basal conditions did not influence body mass in female or male animals, in agreement with previous studies of healthy and diseased mice after varying lengths of treatment (21, 22, 33, 34). However, although muscle strength was unaffected by CARM1 inhibition, in contrast, motor performance, movement behaviors, and exercise capacity were impacted, particularly in male mice. Taken together, these data are not surprising because *1*) CARM1 is critical for maintaining homeostatic cell biology in several, if not all tissues, including the heart and neurons (6, 35), which in addition to skeletal muscle are important for physical performance; *2*) Male CARM1 mKO mice exhibit reduced mobility and run time to exhaustion (8); *3*) CARM1 protein and mRNA content were higher (*P* = 0.07; data not shown) in the muscle of male mice compared with female mice, although CARM1 activity was similar between sexes; and *4*) differing sex hormone profiles and known sex-specific influences of CARM1 (36–42) likely play a role in the disparate whole body physiological responses between males and females. In the context of short-term, pharmacological CARM1 inhibition as cancer therapy (19), a temporary lapse in muscle function and performance may be a negligible side effect for an efficacious treatment.

Recent studies have investigated CARM1 during conditions of neurogenic skeletal muscle atrophy (15, 17, 18). Our results align with previous data from Ref. 18, as we observed no effect of DEN on CARM1 protein content in the GAST muscle, but 50% greater *CARM1* mRNA content (*P* < 0.05; data not shown) 7 days post-DEN in both EZM- and Veh-treated mice. Consistent with this, Stouth et al. (15, 17) observed a significant increase in CARM1 protein content in the TA muscle after 7 days of neurogenic muscle disuse. This nuanced response to DEN between muscles may be due to different CARM1 expression patterns observed across specific skeletal muscles. For instance, in the GAST, PRMT3, and PRMT5 appear to be equally expressed with CARM1, whereas in the SOL, CARM1 is the most abundant PRMT (13), although these analyses have not been completed in the TA. We found that CARM1 protein methylation activity was significantly greater in DEN relative to CON muscle as previously reported (15), and as expected, this effect was completely blunted with EZM treatment. However, when investigating the impact of DEN on CARM1 transcriptional target FGFBP1 (8), we observed the opposite effect whereby DEN markedly reduced FGFBP1 levels. This finding is concordant with earlier work by Taetzsch et al. (43) who observed reduced FGFBP1 expression in skeletal muscles from aged mice and animals with amyotrophic lateral sclerosis. Taken together, these data lead us to speculate that the transcriptional function of CARM1 in atrophying skeletal muscle may be decoupled from its protein arginine methyltransferase activity. The reduction of FGFBP1 in response to DEN is therefore likely influenced by several factors, including but not limited to CARM1, however, more work is necessary to accurately define this mechanism.

It has previously been shown that the absence of CARM1 in skeletal muscle partially attenuates the loss of muscle mass and size following DEN (15, 18). We, therefore, hypothesized that pharmacological CARM1 inhibition would also mitigate neurogenic muscle atrophy. However, neither neurogenic disuse-evoked loss of skeletal muscle mass and myofiber type-specific CSA, nor fiber type composition were attenuated by CARM1 inhibition, even though EZM treatment was effective at blunting the DEN-induced increase in CARM1 methylation activity in skeletal muscle. Moreover, the response to DEN of the master regulator of mitochondrial biogenesis, PGC-1α, as well as the induction of several representative, canonical markers of the atrophy, autophagy, and muscle protein synthesis programs, was unchanged with CARM1 suppression. We postulate that these disparate findings between CARM1 mKO and EZM treatment are at least partly due to the lack of CARM1 in mKO muscles from inception and ensuing adaptation across the lifespan versus global CARM1 inhibition only in adult mice. For instance, CARM1 is important for embryonic development (44) and the optimal functioning of muscle progenitor cells (45, 46), which likely contributes to reduced muscle mass in CARM1 mKO compared with age-matched WT mice (8, 15) that was not observed with the CARM1 inhibitor course utilized in the current study. In support of this, using a unilateral TA-specific CARM1 knock down (KD) in mice at 4 wk before DEN, Liu et al. (18) observed no difference in muscle mass between the CARM1 KD TA and the contralateral control TA muscle at baseline or 2 wk post-DEN but found attenuation of mass loss in the CARM1 KD muscle after 4 wk of DEN. Thus, we hypothesize that pharmacological CARM1 inhibition could have a similar blunting effect on muscle atrophy following a longer duration of DEN. In our analyses, we combined male and female data in the DEN experiments because sex-specific effects of CARM1 inhibition were not observed under these conditions, which suggests the robust impact of relatively short-term DEN may supersede more modest effects of sex. Interestingly, pharmacological CARM1 inhibition contributed to a greater DEN-induced reduction in mitochondrial oxidative protein complexes, specifically complexes III and IV. Previous work showed that CARM1 mKO mice have a greater number of grossly distorted mitochondria (8). Taken together, CARM1 may play a role in mitochondrial biology in skeletal muscle, and this deserves further investigation given the central role of the organelle in muscle function.

We recognize several limitations in this study. First, since EZM globally inhibits CARM1 throughout the body, we cannot determine how pharmacological CARM1 suppression affects skeletal muscle biology independent of the response in other tissues. For example, although we did assess the impact of EZM administration on CARM1 methyltransferase activity in the liver (see results), it remains unclear how the “off-target” effects of the compound in other organs influenced our results. The observed effect of CARM1 suppression on exercise capacity may be due, at least in part, to reduced CARM1 activity in one or more tissues not studied. In addition, because food intake was not measured, it is unclear how potential differences in this variable between Veh and EZM groups may have influenced our findings. Given that CARM1 is expressed in the gastrointestinal system (47), CARM1 inhibition may have caused changes to gastrointestinal function to alter consumption and absorption, endocrine signaling, and/or whole body metabolism. Finally, the molecular mechanisms that regulate skeletal muscle mass and atrophy in humans do not entirely correspond with those observed in mice (48). Thus, further research is needed to understand the role of CARM1 in human muscle atrophy.

In conclusion, our data demonstrate that pharmacological inhibition of CARM1 is effective at reducing the methyltransferase activity of the enzyme in skeletal muscle. Although blocking CARM1 minimally affected body mass or muscle mass, we found a sex-specific decrease in exercise capacity and muscular endurance such that female mice were less impacted by CARM1 suppression. In addition, DEN increased CARM1 activity in skeletal muscle of Veh-treated mice, but this was completely blunted by EZM administration. However, the inhibition of CARM1 had no effect on muscle mass, fiber-specific CSA, and atrophy-related signaling following 7 days of DEN, in contrast to previous findings in CARM1 mKO mice (15, 18). Future studies should use an inducible CARM1 mKO model to address this discrepancy between shorter-term, global pharmacological CARM1 inhibition and the lifelong mKO model. Importantly, despite the modest effects on performance by pharmacologically blocking CARM1, our results indicate that investigations into the use of CARM1 inhibition as a treatment for cancer (19–22, 49–51) and its related sequelae (e.g., cachectic muscle wasting) should continue.

## DATA AVAILABILITY

Data will be made available upon reasonable request.

## GRANTS

This work was funded by the Natural Sciences and Engineering Research Council of Canada (NSERC), the Canada Research Chairs program, and the Ontario Ministry of Economic Development, Job Creation and Trade (MEDJCT). E.K.W. is an interdisciplinary fellow of the Canadian Frailty Network and an NSERC Canada Graduate Scholarship—Master’s program scholar. S.Y.N., D.W.S., and T.L.v. are NSERC Doctoral program scholars. A.I.M. is an Ontario graduate scholarship recipient. V.L. is the Canada Research Chair (Tier 2) in Neuromuscular Plasticity in Health and Disease and is a MEDJCT Early Researcher.

## DISCLOSURES

No conflicts of interest, financial or otherwise, are declared by the authors.

## AUTHOR CONTRIBUTIONS

E.K.W. and V.L. conceived and designed research; E.K.W., S.Y.N., A.I.M., D.W.S., and T.L.v. performed experiments; E.K.W., S.Y.N., A.I.M., D.W.S., T.L.v., A.L.S., and V.L. analyzed data; E.K.W. and V.L. interpreted results of experiments; E.K.W. prepared figures; E.K.W. drafted manuscript; E.K.W. and V.L. edited and revised manuscript; E.K.W., S.Y.N., A.I.M., D.W.S., T.L.v., A.L.S., and V.L. approved final version of manuscript. 
